# *Editorial Commentary*: Step on the GAS: Are We Almost There for Clindamycin and Intravenous Immunoglobulin?

**DOI:** 10.1093/cid/ciu307

**Published:** 2014-04-29

**Authors:** Lionel K. K. Tan, Shiranee Sriskandan

**Affiliations:** Section of Infectious Diseases and Immunity, Imperial College London, United Kingdom

**Keywords:** streptococcal, clindamycin, immunoglobulin, mortality, necrotizing fasciitis

**(See the Major Article by Carapetis et al on pages 358–65.)**

Recent outbreaks of scarlet fever [1], and increased maternal deaths due to invasive streptococcal disease [2] are poignant reminders that group A *Streptococcus* (GAS) remains a global threat to human health. Thankfully, GAS remains susceptible to penicillin, which is usually sufficient for the majority of patients infected with GAS. However, what to do in the minority of patients who develop severe invasive GAS (iGAS), typified by streptococcal toxic shock syndrome (STSS) and necrotizing fasciitis, remains an enigma. The questions facing treating physicians are what adjunctive therapy should be administered, and at what point during the course of the illness? Two particular therapies for severe iGAS infection have been debated over the last 2 decades: clindamycin and intravenous immunoglobulin (IVIG). The other area of ambiguity is whether clinicians should provide antibiotics for close contacts of patients with iGAS. Secondary iGAS may have devastating consequences; however, there is much variation in the recommendations for antibiotic prophylaxis. A study by Carapetis et al [3], in the current issue of *Clinical Infectious Diseases*, hopes to clarify these controversies. The authors admirably set out to answer 3 questions: (1) Does the addition of clindamycin to a β-lactam antibiotic improve mortality? (2) Does additional IVIG improve mortality? (3) Does the risk of secondary iGAS in close contacts justify use of prophylaxis?

The mortality from severe iGAS associated with shock, even when penicillin is used, is reported to be between 30% and 80%, with notable international variation [4–6]. Clindamycin has been shown in vitro to inhibit synthesis of streptococcal superantigens that circulate in STSS [7], and in a mouse model of GAS myositis, clindamycin enhanced survival of animals [8]. However, human clinical studies have been limited to retrospective case analyses with their inherent bias. These have demonstrated either favorable outcomes associated with clindamycin treatment in iGAS [9] and necrotizing fasciitis [10], or, alternatively, no association between clindamycin use and survival [11].

Intravenous human immunoglobulin is able to neutralize circulating superantigens and enhance bacterial clearance [12], providing a rationale for its use in STSS. Concerns regarding biological safety and cost have limited IVIG supply in countries such as the United Kingdom and, in contrast to other indications such as immune thrombocytopenic purpura, there are no adequately powered trials supporting IVIG use in STSS. One multicenter, randomized, double-blind, placebo-controlled trial failed to recruit sufficient patients and, due in part to an unexpectedly low mortality rate in the control arm, was underpowered to achieve statistically meaningful outcome data [13]. Inherent logistical difficulties of trials for rapidly fatal but rare acute infections mean that observational studies could provide the best evidence. One cohort study reported significantly reduced mortality in STSS patients treated with IVIG, but used historical controls with high mortality [4]. A more recent retrospective analysis focused on children with a control mortality of only 4.5%, suggesting that patients may have been at the less severe end of the iGAS spectrum [14].

The data presented in the current study appear to favor the 2 interventions, with an odds ratio of 0.28 (95% confidence interval [CI], .10–.80) in favor of clindamycin, and an odds ratio of 0.12 (95% CI, .01–1.05) in favor of IVIG in addition to clindamycin. However, as the study was based on a fixed period of active surveillance in the state of Victoria in Australia undertaken 10 years ago [15], it lacks the power to unequivocally answer these questions.

During the group's earlier analysis, cases of iGAS were ascertained prospectively from an extensive network of laboratories, hospitals, and primary care practitioners [15]. For the recent study, cases of severe iGAS were identified by the investigators from submitted data collection forms, although investigators relied on treating clinicians to diagnose severe iGAS cellulitis and necrotizing fasciitis, potentially leading to over- or underreporting of cases. Furthermore, any effect of polymicrobial involvement in these 2 conditions would be difficult to determine. Eighty-four patients with severe iGAS were identified, and 63% were admitted to a major teaching hospital. Whether these patients were de novo admitted to a teaching hospital or transferred due to severity of disease requiring further specialist input is unstated. However, once at the teaching hospital, presumably being treated by infection specialists, patients were significantly more likely to receive clindamycin. This is unsurprising, as surveys suggest that clindamycin is generally recommended by infectious disease physicians for patients with severe iGAS, despite the limited evidence [16].

Patients treated with clindamycin had half the mortality rate compared with patients who did not receive clindamycin, and the authors observe that this is despite clindamycin-treated patients having more severe disease, as judged by STSS, admission to the intensive care unit (ICU), and length of stay. This observation should be interpreted with caution. First, although STSS was used as a marker of severity, the inclusion of rash and soft tissue necrosis as potential STSS criteria means that a diagnosis of STSS does not necessarily indicate additional organ dysfunction. Second, the clindamycin-treated patients were of significantly younger age, a known predictor of improved survival in iGAS [5, 6, 11]; indeed, 86% of fatalities in the current study were among patients aged ≥60 years. ICU admission and length of stay may be less reliable markers of disease severity than objective physiological scoring systems. Instead, they may represent markers of early severe sepsis recognition, comorbidities, or interventions, which are independent of initial disease severity. Older age may be a barrier to ICU admission, if perceived as a predictor of poorer outcome, additionally the association between clindamycin administration and pseudomembranous colitis may influence the prescribing behavior of clinicians, leading to potential bias if older patients do not receive this drug.

The authors adjust for these confounding factors (age ≥60 years, STSS, type of hospital, and ICU admission) in multivariate analysis, and although the odds ratios remained suggestive of a benefit from clindamycin treatment, all the 95% CIs stubbornly crossed 1.0. Hence, the effect of adjunctive clindamycin therapy on mortality, independent of treatment decisions based on individual patient factors such as age, still lacks clarity. Perhaps a case-control study design with groups matched for age and disease severity at presentation would have helped to address this problem. Furthermore, no mention is made of the impact of surgery in addition to clindamycin, or the timing of initiation of antimicrobial therapy, both of which are factors that may influence outcome.

Whereas the statistical interpretation of the role of clindamycin on outcome may be hindered by a number of confounding factors, the small number of patients (n = 14) receiving IVIG in this study strikingly limits this part of the analysis. It has been calculated that a sample size of 70 would be required to achieve a statistically significant mortality reduction from 60% to 40% [16]. Perhaps inevitably, only patients receiving clindamycin were treated with IVIG, thus making it difficult to clearly define the independent benefit of IVIG. Indeed, at the time of the original study, IVIG was not easily available in Victoria [15]. Notably, IVIG-treated patients were less likely to be immunocompromised or have an underlying chronic condition, suggesting an inherent bias in the patients selected to receive this adjunctive therapy. These factors may influence the case fatality rate; chronic medical conditions adversely effect outcome in severe GAS infections [5].

In a separate analysis, the investigators report an incidence rate of secondary iGAS that is >2000-fold higher among household contacts than among the general population, favoring the use of antibiotic prophylaxis. The investigation required enumeration of total household contacts at risk, identified via a retrospectively administered postal questionnaire. Although undoubtedly an impressive undertaking, the exclusion criteria for the questionnaires meant that only 95 responses (38%) were received from the 251 index patients who had been living at home. Extrapolating the mean number of household contacts per index patients from the subset that responded resulted in a calculated risk ratio with a very wide 95% CI (413–5929). This imprecision is perhaps expected, as there was a range of 0–12 household contacts per index patient from questionnaire responders. Whether antimicrobial prophylaxis can reduce household risk remains unknown. Although it has been administered in some countries, the resulting benefit has not been reported, and the optimum choice and duration of antimicrobial are unclear.

Although this study is certainly not definitive, it adds to the growing literature that supports use of clindamycin in severe iGAS; however, the adjunctive role of IVIG remains unclear, although there is some suggestion of benefit. Increasing clindamycin resistance observed in recent outbreaks [1] may limit the relevance of this study in some regions, necessitating evaluation of alternative protein synthesis inhibitors. While infectious disease specialists appear to have the appetite for a properly controlled trial of IVIG [16], the daunting number of patients needed would require a truly global effort and be technically challenging. Instead, accumulating evidence from commendable studies such as this will be required to inform our treatment decisions in severe iGAS.
